# Impact of on-site pharmacists in residential aged care facilities on the quality of medicines use: a cluster randomised controlled trial (PiRACF study)

**DOI:** 10.1038/s41598-023-42894-5

**Published:** 2023-09-25

**Authors:** Ibrahim Haider, Sam Kosari, Mark Naunton, Theo Niyonsenga, Jane Koerner, Gregory Peterson, Rachel Davey

**Affiliations:** 1 Discipline of Pharmacy, Faculty of Health, University of Canberra, Bruce, ACT 2617 Australia; 2 Health Research Institute, Faculty of Health, University of Canberra, Bruce, ACT 2617 Australia; 3 School of Pharmacy and Pharmacology, University of Tasmania, Hobart, TAS 7005 Australia

**Keywords:** Health care, Geriatrics, Health policy, Health services

## Abstract

Residents of residential aged care facilities (RACFs) have a high prevalence of use of potentially inappropriate medications (PIMs) and resultant medicines-related harm. This study investigated the effect of an on-site pharmacist model on PIMs use and other medication outcomes for residents in RACFs. A multi-facility, non-blind, cluster randomised controlled trial, with randomisation at the facility level, was conducted. Fifteen facilities enrolled and participated in the study, 7 facilities (560 residents) were allocated to the intervention arm and 8 facilities (737 residents) were allocated to the control arm. Each facility in the intervention arm employed an on-site pharmacist for 12 months to perform medication management activities as part of an interdisciplinary care team. The primary outcome was the proportion of residents taking at least one PIM according to the 2019 Beers® Criteria. Using generalised linear mixed-effects models, accounting for confounders and clustering, there was a significant reduction in the proportion of residents prescribed at least one PIM (odds ratio 0.50, 95% confidence interval, 0.335–0.750; p = 0.001) in the intervention arm. There were also significant decreases in the Anticholinergic Cognitive Burden scale and chlorpromazine equivalent daily dose of antipsychotics. The on-site pharmacist intervention significantly improved the appropriateness of medicines use in RACFs.

## Introduction

Older people living in residential aged care facilities (RACFs) (also known as “long-term care homes” or “nursing homes”) are often on trajectories of physiological and cognitive decline characterised by co-morbidities and the use of multiple medications^1^. They take, on average, 9 to 11 regular medications; this is known as polypharmacy, and it is associated with an increased risk of medication-related problems, adverse drug events, and hospitalisations^2,3^. Studies report that around 95% of aged care residents have at least one medication-related problem^4^, and between 30 and 70% of residents are prescribed one or more potentially inappropriate medications (PIMs)^4^. This has not improved over time, with a recent large study published in 2022 in an Australian RACF setting reporting that 68% of residents were prescribed at least one regular PIM^5^. A meta-analysis of 33 studies found a statistically significant link between hospitalisation and use of PIMs among the elderly^6^. More recently, another meta-analysis of 21 studies showed PIMs to be associated with increased odds of hospital admissions and emergency department visits^7^. PIMs use has also been associated with increased medical costs and a higher risk of adverse outcomes, such as falls, fractures, cognitive decline, and cardiovascular events^8^.

Antipsychotics, sedatives, and medications with high anticholinergic properties are of particular concern amongst the elderly, often causing increased risk of confusion and falls. The use of psychotropics in RACFs was highlighted as a concern in the Australian Royal Commission into Aged Care Quality and Safety report^9^. Antipsychotics are often used to manage behavioural and psychological symptoms of dementia among RACF residents^10^. Australian studies have shown that inappropriate use of antipsychotics is common in RACFs, with over 20% of residents prescribed antipsychotics on a regular basis^11–13^ and often for longer durations than recommended^14^ leading to increased risk of hospitalisation, hip fracture, pneumonia, stroke and death^15,16^. Anticholinergic medications have also been associated with functional and cognitive decline^17,18^. The risk of hospitalisation for confusion, delirium and dementia doubles in residents who are taking one or more anticholinergic medication^19^.

Issues such as the inappropriate use of psychotropics medications in RACFs are often complex with many contextual factors. A recent overview of reviews suggested that multi-disciplinary collaboration, along with education and training, may be an effective approach in tacking the issue^20^. A recent review of pharmacists’ practice models in RACFs from Australia, England, and USA showed there was scope for improving the level of collaboration between pharmacists and other health care professionals^21^. A systematic review analysing factors influencing medication safety in RACFs concluded that lack of accessibility to pharmacists and doctors, and poor interdisciplinary collaboration were considered barriers to the quality use of medicines (QUM) in RACFs^22^.

In Australia, pharmacists perform two government-funded clinical services in aged care facilities, the residential medication management review (RMMR) and QUM services. The RMMR has been a useful tool in identifying and resolving medication-related problems^4,23^. However, RMMRs are conducted on a visitation and ad hoc basis, which may limit residents from receiving services in a timely manner when they are needed, for example, during transitions of care. Also, the visiting pharmacists performing RMMRs may not have a thorough understanding of the resident and the multi-disciplinary healthcare team involved in their care^24^. The QUM program funds pharmacists to visit RACFs and provide education and improve medication management at the facility level^25,26^; however, little research has been conducted to assess the effectiveness of this service^27^.

In view of high rates of inappropriate medicine use despite the existing limited pharmacy services, the Pharmacist in Residential Aged Care Facilities (PiRACF) cluster randomised trial aimed to evaluate a new model in which on-site pharmacists perform medication management activities to improve QUM. On-site pharmacists worked in collaboration with the interdisciplinary care team in RACF to conduct day-to-day medication management tasks, both at the resident level such as medication reviews and at the RACF level to improve policies and practice. This trial assessed the impact of the model on medication appropriateness in RACFs^28^.

## Methods

### Study design

The PiRACF study was a cluster-randomised controlled trial^28^. It was conducted in 15 RACFs in the ACT, Australia. Participating RACFs were randomly allocated, into either a control or intervention group. RACFs in the control arm continued the ‘usual care’, that included receiving government-funded RMMR and QUM services from visiting pharmacists^21^. The study was conducted from April 2019 to December 2021. RACFs in the intervention arm each employed an on-site pharmacist for 2 or 2.5 days per week as member of their care team and continued to receive usual care services A study protocol has been published^28^.

### Ethics statement

Consent to participate in the study was gained at the facility level, rather than the resident level, given the impracticalities of gaining informed consent from a large sample of individual residents, many of whom are likely to have cognitive impairment; there was a low risk to participants and actions were taken to protect participant privacy. Residents were able to opt out of having their data included in the study, and the process on how to do this was provided to residents and families. The trial was approved by Human Research Ethics Committees at the University of Canberra (HERC:2007), ACT Health (2019/ETH13453) and Calvary Public Hospital Bruce (30-2019).

The trial was registered with the Australian New Zealand Clinical Trials Registry (ANZCTR) (ACTRN: ACTRN12620000430932 on 01/04/2020). All experiments were performed in accordance with relevant guidelines and regulations. Findings are reported in accordance with the extension of the Consolidated Standards of Reporting Trials (CONSORT) statement to cluster randomised controlled trials^29^.

### Randomisation and recruitment

Randomisation was performed at the facility level. RACFs were stratified by size of facility and randomised into either the intervention or control group through computer-generated allocation by an independent person external to the research team. All RACFs in the ACT, Australia, that met the inclusion criteria were invited to participate in this study. Due to the nature of the intervention, participants were not blinded. Permanent residents of participating RACFs were included unless they requested to opt out. New residents entering RACFs after the baseline data collection that remained at the RACFs until the end-point data collection, were also included. Respite (non-permanent) residents were excluded. Only residents who were aged 65 years or over were included in the data analysis.

### Intervention

Each participating intervention RACF directly employed a pharmacist as a facility staff member reporting to the RACF manager, to work on-site for 2 to 2.5 days per week for 12 months. Salaries for pharmacists were funded by the research grant. The intervention (model of care) was informed by the findings of a pilot study and discussion with RACF managers, general practitioners (GPs), pharmacists, and a consumer representative who participated in the pilot^30–32^. Training and support were provided to pharmacists prior to starting their role and during the period of the intervention^28^. RMMR and QUM services were conducted by RACF usual contractors in both study arms and did not impact the services provided by the on-site pharmacists. On-site pharmacists performed activities including medication reviews and medication reconciliation at transitions of care; clinical audits to identify those at highest risk of medication-related problems; group and individual education with staff, residents, and families; and improving facility’s medication management policies. A list of pharmacist activities in the model of care was detailed in the protocol^28^. On-site pharmacists self-reported their daily activities through an online diary.

### Data collection

Demographic data, drug chart information including diagnoses and medication data were collected from RACFs by the PIRACF study research team at two timepoints, baseline and after 12 months. Data was collected by the research team from the electronic or paper records at the RACF. The 2nd timepoint data were collected 12 months after the start of the pharmacist intervention (end of intervention).

### Outcomes measures

The primary outcome measure was the proportion of residents who were taking at least one PIM according to the American Geriatrics Society Beers® 2019 criteria^33^. The Beers® criteria were modified in this study to fit the Australian setting to include medications that are available in Australia^8^. The use of PIMs has often been used a marker for QUM in RACFs^6,34–36^. The first author (I.H.) applied the Beers® Criteria to residents’ medications with ongoing review by the chief investigators. The unit of analysis for medication-related outcomes was at the resident-level.

Secondary outcomes included the proportion of residents who were prescribed at least one psychotropic medicine (defined as antipsychotics and benzodiazepines in the absence of major psychiatric disease or epilepsy), residents’ daily dose of psychotropic medicines (measured as chlorpromazine and diazepam equivalent daily dose per resident)^37,38^, residents’ mean Anticholinergic Cognitive Burden (ACB) scale^39^, the number of regular medications, and the proportion of residents who had complete documentation of drug allergies or adverse drug reactions in their RACF records.

### Statistical analyses

Descriptive statistics included mean and standard deviation for numeric variables and proportion for categorical variables. Mann–Whitney-U tests and Chi-square were used for unadjusted comparison of (continuous and categorical, respectively) baseline characteristics between control and intervention arms.

This study used generalised linear mixed-effects models (GLMMs) to compare outcome variables between intervention and control groups at baseline and endpoint. Logistic GLMM was used for binary outcome variables (e.g., taking at least one PIM: yes/no), Poisson was used for discrete count variables (e.g., number of regular medications), and gamma distributions were used for continuous positively skewed variables (e.g., daily dose of psychotropic medicines). The models adjusted for potential confounders (age, gender, Charlson Comorbidity Index (CCI), presence of other educational intervention^40^, diagnosis of dementia and number of regular medicines), and accounted for clustering of residents within RACFs and repeated observation within residents. The intra-cluster correlation coefficient (ICC) at baseline was calculated for the primary outcome^41^. For each outcome variable, the model estimated main effects of group (control or intervention), time (baseline and endpoint) and the interaction group by time effect (group*time). The estimated coefficient associated to the interaction term reflects the difference in changes over time by study arms, also known as the difference-in-difference approach between group effect (intervention or control) and time effect (baseline and intervention)^42^. Results are reported with 95% confidence intervals (CI). Missing values were uncommon (less than 1%) and a listwise deletion was applied. Data analysis was conducted using the Statistical Package for Social Sciences (SPSS, version 27.0; IBM Corp. Armonk, USA). Any result with an associated probability value less than 5% (*p* < 0.05) was considered statistically significant.

## Results

### Study population

**Figure 1:**
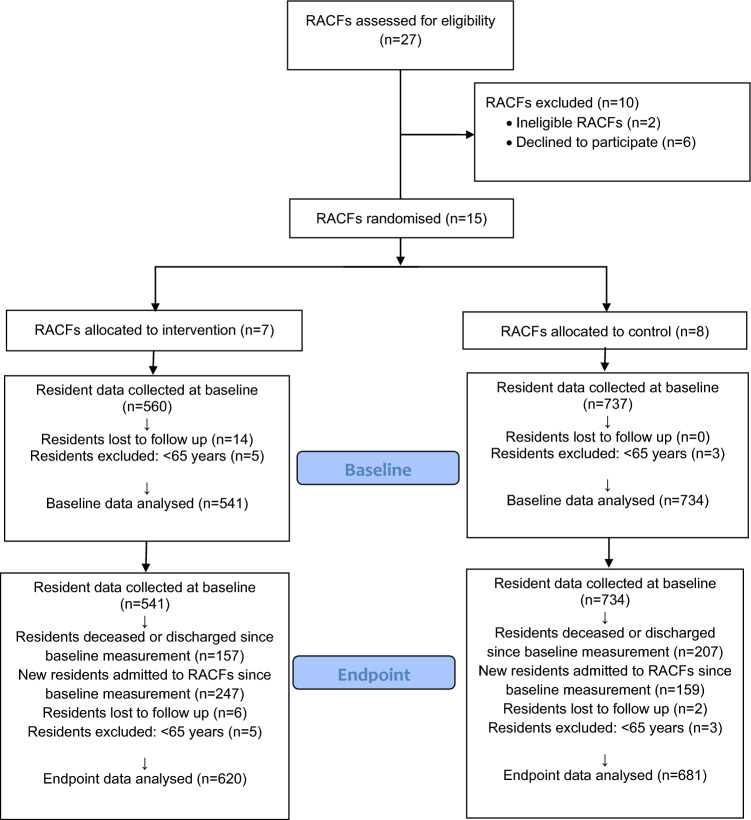
Study participants flowchart.

Fifteen RACFs enrolled and participated in the study. After randomisation, 7 RACFs (560 residents) were allocated to the intervention arm and 8 RACFs (737 residents) were allocated to the control arm. Twenty-two residents were lost to follow-up and 16 residents were excluded because they were under 65 years of age, where the Beers® Criteria are not applicable. Overall, 1275 residents from the baseline and 1301 residents from the endpoint were included in the final analysis. The flow diagram of the study population is presented in Fig. 1.

The baseline characteristics of participating residents were similar between groups, although the control group had slightly more females and had higher percentage of residents diagnosed with dementia (Table 1). The demographics of participating residents were compared to Australian national data for RACF residents and is presented in Table 2.

**Table 1:** Baseline characteristics of study participants (RACF residents).

Characteristic	Control (%) (n = 734)	Intervention (%) (n = 541)
Age (years)		
65–69	21 (2.9)	4 (0.7)
70–74	63 (8.6)	30 (5.5)
75–79	69 (9.4)	48 (8.9)
80–84	124 (16.9)	89 (16.5)
85 +	457 (62.3)	370 (68.4)
Sex		
Male	271 (36.9)	165 (30.5)
Female	463 (63.1)	376 (69.5)
Aboriginal and Torres Strait Islander status		
Yes	3 (0.4)	3 (0.6)
No	717 (97.7)	530 (98.0)
Preferred language		
English	583 (79.4)	461 (85.2)
Other	151 (20.6)	80 (14.8)
Number of regular medications		
Less than 5	37 (5.0)	40 (7.4)
5–9	202 (27.5)	131 (24.2)
10 or more	495 (67.4)	370 (68.4)
Charlson comorbidity index (CCI)		
0	89 (12.1)	56 (10.4)
1	202 (27.5)	144 (26.6)
2	158 (21.5)	114 (21.1)
3 +	285 (38.8)	227 (42.0)
Dementia diagnosis		
Yes	365 (49.9)	215 (41.8)
No	371 (50.1)	315 (58.2)
Number of PIMs per resident		
0 PIMs	245 (33.4)	159 (29.4)
≥ 1 PIMs*	487 (66.6)	382 (70.6)
≥ 2 PIMs*	257 (35.0)	192 (35.4)
≥ 3 PIMs*	95 (12.9)	92 (17.0)

PIMs = Potentially Inappropriate Medications, * PIMs categories ≥ 1 are inclusive categories (i.e., ≥ 1 PIMs includes data shown in ≥ 2 PIMs and ≥ 3 PIMs and thus % values do not sum to 100%).

**Table 2:** Comparison between PiRACF study sample characteristics (at baseline) and national data.

Variable	PiRACF study N = 1275	National data* N = 179,993	P value
Age (years) do the same a GEN data			**0.001**
65–69	25 (2%)	6290 (3.5%)	
70–74	93 (7.3%)	13,145 (7.3%)	
75–79	117 (9.2%)	20,343 (11.3%)	
80–84	213 (16.7%)	32,369 (18%)	
Equal to or over 85 years	827 (64.9%)	107,840 (60%)	
Sex			0.513
Male	436 (34.2)	59,983 (33.3%)	
Female	839 (65.8)	120,004 (66.7%)	
Aboriginal and Torres Strait Islander status			0.167
Yes	6 (.3)	1562 (0.9%)	
No	1247 (97.7)	178,300 (99.1%)	
Preferred language			** < 0.001**
English	1044 (81.9)	160,669 (90.8%)	
Others	231 (18.1)	16,374 (9.2%)	

*Residents under 65 years were excluded from the national data. Statistically significant differences between PiRACF study and nationally representative data shown in bold text.

### Impact of intervention on the primary outcome

Table 3 shows descriptive results at baseline and endpoint for each of the study outcomes. The proportion of residents who were prescribed at least one regular PIM in the intervention group reduced from 70.6% [interquartile interval 66.6–74.4%] at baseline to 60.8% [interquartile interval 56.8–64.7%] at endpoint, with little change in the control group over the study period (Table 3). Table 4 presents a summary of the results for the primary and secondary study outcomes. This is presented as the two-way interaction effect of intervention at the endpoint (i.e., intervention arm * Endpoint) for both unadjusted models and after adjusting for clustering and potential confounders. Supplementary Tables 1–7 present the full details of each model with the main effects, interactions effects, and confounders considered in adjusted models. In the GLMM logistic model for the primary outcome when after adjusting for confounders, residents in the intervention group had halved the odds of having at least one prescribed PIM over the period of the study, when compared to the control group (odds ratio 0.50, 95% CI: 0.335–0.750; *P* = 0.001). Clustering of the primary outcome was lower than anticipated at baseline (ICC = 3.17%, 95% CI 1.0–9.64%).

**Table 3:** Descriptive data for medication-related outcomes at baseline and endpoint.

Outcome*	Timepoint	Control (95% CI) n (baseline) = 734 n (endpoint) = 681	Intervention (95% CI) n (baseline) = 541 n (endpoint) = 620
Proportion of residents who were prescribed 1 + regular PIMs	Baseline	66.6% (63.1–70.0)	70.6% (66.6–74.4)
	Endpoint	67.0% (63.3–70.5)	60.8% (56.8–64.7)
Mean ACB scale	Baseline	1.2 (1.1–1.3)	1.2 (1.1–1.4)
	Endpoint	1.1 (1.0–1.3)	0.9 (0.8–1.1)
Proportion of residents prescribed 1 + antipsychotic or benzodiazepine	Baseline	25.1% (22.0–28.4)	24.6% (21.0–28.4)
	Endpoint	23.8% (20.6–27.2)	18.4% (15.4–21.7)
Mean chlorpromazine equivalent daily dose per resident (mg)	Baseline	15.4 (11.9–19.1)	12.5 (9.0–16.0)
	Endpoint	15.2 (11.2–19.1)	8.64 (6.1–11.3)
Mean diazepam equivalent daily dose per resident (mg)	Baseline	0.8 (0.7–1.0)	0.8 (0.5–1.0)
	Endpoint	0.5 (0.3–0.6)	0.4 (0.3–0.6)
Proportion with complete ADR documentation	Baseline	97.4% (96.0–98.4)	95.6% (93.5–97.1)
	Endpoint	99.1% (98.1–99.7)	98.2% (96.8–99.1)
Mean number of regular medicines per resident	Baseline	9.9 (9.5–10.2)	10.0 (9.6–10.4)
	Endpoint	9.1 (8.8–9.5)	9.6 (9.3–10.0)

*Proportions presented as % and continuous variables as means.

*ACB* Anticholinergic Cognitive Burden, *ADR* Adverse Drug Reaction, *mg* milligram, *PIM* Potentially Inappropriate Medication.

**Table 4:** Summary of results from generalized linear mixed-models of medication-related outcomes in the PiRACF study (reference category: controls at baseline) (1) the unadjusted model controlled for the clustering structure of the data only. (2) The adjusted model controlled for the clustering structure of the data and adjusted for potential confounders. (3) Main effect of intervention group in comparison to control as reference at baseline. (4) Interaction term (intervention × endpoint).

Outcomes	Unadjusted model^(1)^	p value	Adjusted model^(2)^	p value
Primary outcome: Proportion of residents who were prescribed 1 + regular PIMs				
Control versus intervention at baseline^(3)^ (OR)	1.215 (0.819–1.802)	0.937	1.160 (0.754–1.785)	0.499
(Control vs. intervention at baseline) compared to (control vs. intervention at endpoint)^(4)^ (Odd ratio (OR))	**0.595 (0.414**–**0.855)**	**0.005**	**0.501 (0.335**–**0.750)**	**0.001**
Secondary outcomes: Residents’ ACB scale				
Control vs. intervention at baseline^(3)^ (RR)	1.054 (0.857–1.298)	0.617	1.017 (0.797–1.299)	0.890
(Control vs. intervention at baseline) compared to (control vs. intervention at endpoint) (RR)	**0.832 (0.705**–**0.981)**	**0.028**	**0.800 (0.678**–**0.944)**	**0.008**
Proportion of residents prescribed one or more benzodiazepine or antipsychotic				
Control vs. intervention at baseline (OR)	0.999 (0.736–1.357)	0.996	0.946 (0.628–1.424)	0.790
(Control vs. intervention at baseline) compared to (control vs. intervention at endpoint) (OR)	0.732 (0.487–1.101)	0.134	0.676 (0.439–1.042)	0.076
Mean chlorpromazine equivalent daily dose (mg) per resident				
Control vs. intervention at baseline (β Coeff)	− 0.120 (− 0.345 to 0.106)	0.298	0.006 (− 0.204 to 0.217)	0.953
(Control vs. intervention at baseline) compared to (control vs. intervention at endpoint) (β Coeff)	− 0.198 (− 0.405 to 0.008)	0.060	** − 0.250 (− 0.456 to − 0.043)**	**0.018**
Mean diazepam equivalent daily dose (mg) per resident				
Control vs. intervention at baseline (β Coeff)	0.046 (− 0.195 to 0.288)	0.707	− 0.111 (− 0.308 to 0.085)	0.265
(Control vs. intervention at baseline) compared to (control vs. intervention at endpoint) (β Coeff)	− 0.093 (− 0.400 to 0.214)	0.551	− 0.129 (− 0.428 to 0.170)	0.397
Proportion of residents with complete ADR documentation				
Control vs. Intervention at baseline (OR)	0.766 (0.460–1.276)	0.305	0.813 (0.473–1.398)	0.453
(Control vs. intervention at baseline) compared to (control vs. intervention at endpoint) (OR)	1.127 (0.518–2.450)	0.763	1.109 (0.510–2.408)	0.794
Mean number of regular medicines per resident				
Control versus intervention at baseline (RR)	1.034 (0.927–1.154)	0.547	1.029 (0.974–1.087)	0.558
(Control vs. intervention at baseline) compared to (control vs. intervention at endpoint) (RR)	1.033 (0.978–1.092)	0.243	1.029 (0.974–1.087)	0.313

Significant values are in bold.

OR = odds ratio, RR = rate risk, Coeff = coefficient, ACB = Anticholinergic Burden, ADR = Adverse Drug reaction, PIM = Potentially Inappropriate Medication. Adjusted model includes age, sex, presence/absence of a dementia diagnosis, CCI, number of regular medications, and presence/absence of concurrent NPS MedicineWise intervention. Baseline Control n = 734, Intervention n = 541, Endpoint Control n = 681, Intervention n = 620.

### Impact of intervention on secondary outcomes

Resident’s mean ACB scale decreased from 1.21 at baseline to 0.94 at endpoint in the intervention group, and from 1.21 at baseline to 1.14 at endpoint in the control group. Similarly, models estimating resident’s ACB scale showed a lowered risk of ACB scale with a rate ratio of 0.800 (95% CI: 0.678-0.0944) in the intervention group when compared to the control group over the period of the study. Residents in the intervention group had lower odd of taking one or more benzodiazepines or antipsychotics (odds ratio 0.68, 95% CI 0.439–1.042) over the study period but was not statistically significant (*P* = 0.076). When estimating the effect of the intervention on the doses of residents’ antipsychotics, the chlorpromazine equivalent daily dose showed a statistically significant decrease of 0.250 mg (95% CI − 0.456 to − 0.043) (*P* = 0.018) over the study period. The diazepam equivalent daily dose of benzodiazepines per resident was reduced by 0.129 mg (95% CI − 0.428 to 0.170) but was not statistically significant (*P* = 0.397). Models estimating the effect on the number of regular medications and the proportion of residents with complete ADR documentation showed no statistically significant differences between control and intervention groups (see Supplementary Tables 1–7 for details).

## Discussion

This is the first randomised controlled trial conducted to assess the effects of an on-site pharmacist in residential aged care model on medication appropriateness. The study found a statistically significant reduction in the number of residents using PIMs, residents’ mean anticholinergic burden scale, and chlorpromazine equivalent dose of antipsychotic medications per resident in the intervention group compared to the control. There were no significant changes identified in other outcomes, including number of regular medications, ADR documentation status, the proportion of residents taking one or more benzodiazepine or antipsychotic, and the dose of benzodiazepines between control and intervention groups.

In this study, pharmacists were included as part of each facility’s multi-disciplinary care team to perform resident-level clinical activities, including comprehensive medication reviews, and facility-level activities such as clinical audits and contributing to policies and procedures. This holistic approach to medication management allowed pharmacists to prioritise and resolve medication-related problems as they occurred and mitigate risks through improving organisational systems within the RACF^43–45^. An advantage of integrating pharmacists within the healthcare team of RACF is the potential for improved collaboration and communication, as pharmacists work in proximity with others healthcare team members. In this model pharmacists are part of the team of RACFs as compared to external contractors who visit the RACFs. On-site pharmacists can regularly communicate and collaborate with RACF team members, residents, families, GPs and other RACF health professionals and contribute to the multi-disciplinary care of residents. A study of the activities conducted by OSPs in the PiRACF study showed the wide range of activities conducted by OSPs including clinical medication reviews, education, clinical audits, and quality improvement activities. The OSPs spent a large proportion of their time communicating and collaborating with the GPs, RACFs healthcare team, residents, and their families. Of all recommendations made by OSPs, the rate of prescriber agreement was 51.5%^46^.

The study found a significant decrease in the proportion of residents taking at least one PIM in the intervention group compared to the control group over the study period. According to the adjusted model presented, the odds of having at least one prescribed PIM in the intervention group had halved over the period of the study, when compared to the control group. The use of PIMs has been considered as an indicator for the QUM in healthcare settings^5^, and the presence of PIMs has been linked with significant adverse drug events and hospitalisations among older people^6,8^. In this trial, the control sites followed the usual care where an accredited pharmacist could perform medication reviews (referred to as RMMR), on a visitational basis when they are deemed clinically necessary by prescribers. Visitational services may have limitations, especially during periods of transitions of care where residents are 3 times more likely to experience a medication-related problem^47^. Although the current RMMR service in Australia has demonstrated effectiveness in identifying medication-related issues^23^, a recent longitudinal study of 5576 women in RACFs from 2005 to 2017 determined no evidence for an association between RMMRs and reducing PIMs^48^. Additionally, a recent study showed that only 1 in 5 residents receive an RMMR in the 3 months after being admitted to an RACF in Australia^49^. Australian QUM services aim to improve medicine-related practices in RACFs but limited evidence is available for their effectiveness^27^. The integrated pharmacist approach in RACFs may enhance medication management through improved interdisciplinary collaboration and goes beyond the existing RMMR and QUM services.

The study observed a statistically significant reduction of residents’ anticholinergic drug load in the intervention group. Older residents are particularly vulnerable from the detrimental effects of anticholinergic medications, such as cognitive impairment, constipation, and behavioural disturbances^50^. The use of medications with anticholinergic activity has been linked with an increased risk of falls in older adults^51^, and the Beers® Criteria recommend avoiding the use of highly anticholinergic medications in the elderly^33^. According to a systematic review of pharmacist interventions to improve anticholinergic prescribing practice in older people, medication reviews and education were the most commonly used interventions^52^. A systematic review found that pharmacist-led interventions that involved collaboration with medical practitioners reduced anticholinergic drug burden more than other interventions^45^. A qualitative systematic review found working in silos resulted in low motivation to reduce anticholinergic use, while enablers of successful anticholinergic burden reduction involved good communication with patients, carers, and other healthcare professionals^40^. The nature of the on-site pharmacist intervention enables improved collaboration with other healthcare professionals as well as residents which may facilitate the reduction of residents’ ACB.

The proportion of residents taking psychotropics, defined as one or more benzodiazepines or antipsychotics, had a reduction in the intervention arm when compared to the control but the difference was not statistically significant. While the dose of benzodiazepines didn’t show a reduction, notably, the dose of antipsychotics after adjusting for potential confounders, showed a statistically significant reduction in the intervention arm. This finding suggests the pharmacists were able to initiate reduction in dose of antipsychotics but may have not been able to achieve cessation over the period of the intervention, as the process requires dose tapering and monitoring^53^.

Polypharmacy is prevalent amongst residents in residential age care. In the RACF setting polypharmacy is often defined as the use of nine or more regular medications^54,55^. The average number of regular medications for each resident in this cohort ranged between 9 to 10 regular medications. This study showed no significant effects on the number of regular medications when compared between intervention and control over the period of study. While high levels of polypharmacy have been associated with medication-related problems, hospital admission and falls^56^, there may be a clinically appropriate need for polypharmacy in some residents with high level of multimorbidity. Balancing the risk of undertreatment and reducing polypharmacy can be complex, and further research may be needed to explore barriers in addressing polypharmacy by on-site pharmacists. The proportion of residents who had their allergy and ADR status documented was recorded and was found to be high at baseline with little scope for improvement. Allergies and ADRs have the potential to cause significant harm and hospital admissions^3^, and on-site pharmacists need to pay attention to complete ADR documentation in their roles.

The study had the following limitations. Due to COVID-19 we could not assess the effect of the on-site pharmacist in RACF model on medication round time and inappropriate administration of dosage forms, as was initially planned. The Beers criteria tool was used as the primary outcome of the study to identify the use of potentially inappropriate medications amongst residents in RACFs. While the Beers Criteria tool is a useful proxy for QUM, it does not assess the underuse of helpful medications. All participating facilities in the study were in the Australian Capital Territory (ACT) Australia which is a metropolitan area. A careful analysis of the contextual differences should be considered when generalising to other regions in Australia and beyond, such as regional and rural areas. Future studies could assess the longer-term impact of OSP in RACF model on resident health outcomes and include economical analysis for the wider roll out of this model.

## Conclusion

The on-site pharmacist intervention was successful in reducing PIMs, anticholinergic cognitive burden, and the dose of antipsychotics in RACF residents, suggesting that the on-site pharmacist in RACF model improves medication management. The findings strengthen the case for wider implementation and integration of pharmacists into multi-disciplinary care teams in RACFs to help improve residents’ quality use of medicines.

### Supplementary Information

Supplementary Tables.

## Data Availability

The datasets generated and/or analysed during the current study are not publicly available due to the limits subscribed to in the human research ethics committee application but are available from the corresponding author on reasonable request.

## References

[1] Wilson NM, March LM, Sambrook PN, Hilmer SN (2010). Medication safety in residential aged-care facilities: A perspective. Therap. Adv. Drug Saf..

[2] Shah BM, Hajjar ER (2012). Polypharmacy, adverse drug reactions, and geriatric syndromes. Clin. Geriatr. Med..

[3] Hajjar ER, Cafiero AC, Hanlon JT (2007). Polypharmacy in elderly patients. Am. J. Geriatr. Pharmacother..

[4] Pharmaceutical society of Australia. *PROGRAM RULES Home Medicines Review*. https://www.ppaonline.com.au/wp-content/uploads/2019/01/HMR-Program-Rules.pdf (2019)

[5] Haider I (2022). Quality use of medicines indicators and associated factors in residential aged care facilities: Baseline findings from the pharmacists in RACF study in Australia. J. Clin. Med..

[6] Xing XX (2019). Associations between potentially inappropriate medications and adverse health outcomes in the elderly: A systematic review and meta-analysis. Ann. Pharmacother..

[7] Weeda ER, AlDoughaim M, Criddle S (2020). Association between potentially inappropriate medications and hospital encounters among older adults: A meta-analysis. Drugs Aging.

[8] Harrison SL (2018). Costs of potentially inappropriate medication use in residential aged care facilities. BMC Geriatr..

[9] Royal Commission into Aged Care Quality and Safety. Final Report. *Royal Commission into Aged Care Quality and Safety.* https://agedcare.royalcommission.gov.au/publications/final-report (2021).

[10] Briesacher BA, Tjia J, Field T, Peterson D, Gurwitz JH (2013). Antipsychotic use among nursing home residents. J. Am. Med. Assoc..

[11] Westaway K (2018). The extent of antipsychotic use in Australian residential aged care facilities and interventions shown to be effective in reducing antipsychotic use: A literature review. Dementia.

[12] Westbury J, Gee P, Ling T, Kitsos A, Peterson G (2019). More action needed: Psychotropic prescribing in Australian residential aged care. Aust. N. Z. J. Psychiatry.

[13] Pont LG, Raban MZ, Jorgensen ML, Georgiou A, Westbrook JI (2018). Leveraging new information technology to monitor medicine use in 71 residential aged care facilities: Variation in polypharmacy and antipsychotic use. Int. J. Qual. Health Care.

[14] Lind EK, Raban ZM, Georgiou A, Johanna I (2019). Duration of antipsychotic medication use by aged care facility residents with dementia. Alzheimer Dis. Assoc. Disord..

[15] Pratt N, Roughead EE, Ramsay E, Salter A, Ryan P (2011). Risk of hospitalization for hip fracture and pneumonia associated with antipsychotic prescribing in the elderly. Drug Saf..

[16] Pratt N, Roughead EE, Ryan P, Salter A (2010). Antipsychotics and the risk of death in the elderly: An instrumental variable analysis using two preference based instruments. Pharmacoepidemiol. Drug Saf..

[17] Cai X, Campbell N, Khan B, Chan C, Boustani M (2013). Long-term anticholinergic use and the aging brain. Alzheimer’s Dementia.

[18] Carrière I (2009). Drugs with anticholinergic properties, cognitive decline, and dementia in an elderly general population. Arch. Intern. Med..

[19] Kalisch Ellett LM, Pratt NL, Ramsay EN, Barratt JD, Roughead EE (2014). Multiple anticholinergic medication use and risk of hospital admission for confusion or dementia. J. Am. Geriatr. Soc..

[20] Wiggin DA, Timmons S, Rukundo A, Walsh KA (2021). Improving the appropriateness of psychotropic prescribing for nursing home residents with dementia: An overview of reviews. Aging Ment. Health.

[21] Haider I (2021). How do pharmacists practice in aged care? A narrative review of models from Australia, England, and the United States of America. Int. J. Environ. Res. Public Health.

[22] Al-Jumaili AA, Doucette WR (2017). Comprehensive literature review of factors influencing medication safety in nursing homes: Using a systems model. J. Am. Med. Dir. Assoc..

[23] Chen EYH (2019). Process, impact and outcomes of medication review in Australian residential aged care facilities: A systematic review. Australas. J. Ageing.

[24] McDerby N, Naunton M, Shield A, Bail K, Kosari S (2018). Feasibility of integrating residential care pharmacists into aged care homes to improve quality use of medicines: Study protocol for a non-randomised controlled pilot trial. Int. J. Environ. Res. Public Health.

[25] Pharmaceutical society of Australia. *Guidelines for pharmacists providing Residential Medication Management Review (RMMR) and Quality Use of Medicines (QUM) services*. https://www.ppaonline.com.au/wp-content/uploads/2019/01/PSA-RMMR-and-QUM-Guidelines.pdf (2017).

[26] Department of Health and Aging. Guiding principles for medication management in residential aged care facilities. https://www.health.gov.au/resources/publications/guiding-principles-for-medication-management-in-residential-aged-care-facilities?language=en (2022).

[27] Sluggett JK, Ilomäki J, Seaman KL, Corlis M, Bell JS (2017). Medication management policy, practice and research in Australian residential aged care: Current and future directions. Pharmacol. Res..

[28] Kosari S (2021). Integrating pharmacists into aged care facilities to improve the quality use of medicine (PiRACF Study): protocol for a cluster randomised controlled trial. Trials.

[29] Campbell MK, Piaggio G, Elbourne DR, Altman DG (2012). Consort 2010 statement: Extension to cluster randomised trials. BMJ.

[30] McDerby N (2020). Residential aged care pharmacist: An Australian pilot trial exploring the impact on quality use of medicines indicators. Medicines.

[31] McDerby N (2019). The effect of a residential care pharmacist on medication administration practices in aged care: A controlled trial. J. Clin. Pharm. Ther..

[32] McDerby NC (2019). Pharmacist-led influenza vaccination services in residential aged care homes: A pilot study. Australas. J. Ageing.

[33] American Geriatrics Society (2019). American geriatrics society 2019 updated AGS beers criteria for potentially inappropriate medication use in older adults. J. Am. Geriatr. Soc..

[34] Harrison SL (2018). Associations between the drug burden index, potentially inappropriate medications and quality of life in residential aged care. Drugs Aging.

[35] Fick DM, Mion LC, Beers MH, Waller LJ (2008). Health outcomes associated with potentially inappropriate medication use in older adults. Res. Nurs. Health.

[36] Ní Chróinín D (2016). Potentially inappropriate medications (PIMs) in older hospital in-patients: Prevalence, contribution to hospital admission and documentation of rationale for continuation. Australas. J. Ageing.

[37] Woods SW (2003). Chlorpromazine equivalent doses for the newer atypical antipsychotics. J. Clin. Psychiatry.

[38] Hoffmann, F. Benefits and risks of benzodiazepines and Z-drugs: Comparison of perceptions of GPs and community pharmacists in Germany. *German Med. Sci. GMS e-J.* **11**, Doc10 (2013). 10.3205/000178 PMC3728643 23904824

[39] Boustani M, Campbell N, Munger S, Maidment I, Fox C (2008). Impact of anticholinergics on the aging brain: A review and practical application. Aging Health.

[40] Stewart C (2021). Barriers and facilitators to reducing anticholinergic burden: A qualitative systematic review. Int. J. Clin. Pharm..

[41] Austin PC, Merlo J (2017). Intermediate and advanced topics in multilevel logistic regression analysis. Stat. Med..

[42] Australian Government: Department of Health and Aged Care. Education program to help improve dementia care. https://www.health.gov.au/news/announcements/education-program-to-help-improve-dementia-care (2021).

[43] Vliek S (2008). Single versus multicomponent intervention in frail elderly: Simplicity or complexity as precondition for success?. J. Nutr. Health Aging.

[44] Campbell AJ, Robertson MC (2007). Rethinking individual and community fall prevention strategies: A meta-regression comparing single and multifactorial interventions. Age Ageing.

[45] Ali S, Salahudeen MS, Bereznicki LRE, Curtain CM (2021). Pharmacist-led interventions to reduce adverse drug events in older people living in residential aged care facilities: A systematic review. Brit. J. Clin. Pharmacol..

[46] Haider I, Kosari S, Naunton M, Koerner J, Dale M, Nizamani S, Davey R (2023). The role of on-site pharmacist in residential aged care facilities: Findings from the PiRACF study. J. Pharm. Policy Pract..

[47] Elliott RA, Booth CJ (2014). Problems with medicine use in older Australians: A review of recent literature. J. Pharm. Pract. Res.

[48] Thiruchelvam K, Byles J, Hasan SS, Egan N, Kairuz T (2022). Impact of medication reviews on potentially inappropriate medications and associated costs among older women in aged care. Res. Soc. Adm. Pharm..

[49] Sluggett JK (2021). Variation in provision of collaborative medication reviews on entry to long-term care facilities. J. Am. Med. Dir. Assoc..

[50] Salahudeen MS, Nishtala PS (2016). Examination and estimation of anticholinergic burden: Current trends and implications for future research. Drugs Aging.

[51] Ruxton K, Woodman RJ, Mangoni AA (2015). Drugs with anticholinergic effects and cognitive impairment, falls and all-cause mortality in older adults: A systematic review and meta-analysis. Br. J. Clin. Pharmacol..

[52] Salahudeen MS (2022). Effectiveness of interventions to improve the anticholinergic prescribing practice in older adults: A systematic review. J. Clin. Med..

[53] Bjerre L (2018). Deprescribing antipsychotics for behavioural and psychological symptoms of dementia and insomnia evidence-based clinical practice guideline. Can. Fam..

[54] Jokanovic N, Tan ECK, Dooley MJ, Kirkpatrick CM, Bell JS (2015). Prevalence and factors associated with polypharmacy in long-term care facilities: A systematic review. J. Am. Med. Dir. Assoc..

[55] Lalic S (2016). Polypharmacy and medication regimen complexity as factors associated with staff informant rated quality of life in residents of aged care facilities: A cross-sectional study. Eur. J. Clin. Pharmacol..

[56] Jokanovic N (2017). Prioritizing interventions to manage polypharmacy in Australian aged care facilities. Res. Social Adm. Pharm..

