# Supplementary material for: Impact of on-site pharmacists in residential aged care facilities on the quality of medicines use: a cluster randomised controlled trial (PiRACF study)
Source: Sci Rep. 2023 Sep 25;13:15962. doi: 10.1038/s41598-023-42894-5 (PMC10519995; doi:10.1038/s41598-023-42894-5)
Supplement: Supplementary file 1 — Supplementary Tables. [file 41598_2023_42894_MOESM1_ESM.docx]

| **Outcome: Proportion with 1+ regular PIMs** | **Unadjusted model**  **Odds Ratio (95% CI)** | **p value** | **Adjusted model**  **Odds Ratio (95% CI)** | **p value** |
| --- | --- | --- | --- | --- |
| **Control or Intervention arms** |  | | | |
| Control | 1.00 | - | 1.00 | - |
| Intervention | 1.215 (0.819 - 1.802) | 0.937 | 1.160 (0.754 - 1.785) | 0.499 |
| **Timepoints** |  | | | |
| Baseline | 1.00 | - | 1.00 | - |
| Endpoint | 1.010 (0.792 - 1.287) | 0.333 | 1.159 (0.887 - 1.516) | 0.280 |
| **Two-way interaction effects** |  | | | |
| Intervention arm*Endpoint | **0.595 (0.414 - 0.855)** | **0**.**005** | **0.501 (0.335 - 0.750)** | **0.001** |
| **Age** | - | - | **0.975 (0.961 - 0.990)** | **0.001** |
| **Sex** |  |  |  |  |
| Female | - | - | 1.00 | - |
| Male | - | - | 0.845 (0.663 - 1.078) | 0.176 |
| **Dementia diagnosis** |  | | | |
| Residents without dementia diagnosis | - | - | 1.00 | - |
| Residents with dementia diagnosis | - | - | 1.030 (0.817 - 1.299) | 0.803 |
| **Total CCI** | - | - | 1.034 (0.965 - 1.107) | 0.343 |
| **Number of regular medications** | - | - | **1.317 (1.275 - 1.361)** | **<0.001** |
| **NPS MedicineWise intervention** |  | | | |
| No Intervention | - | - | 1.00 | - |
| Intervention | - | - | 1.011 (0.580 - 1.764) | 0.968 |

**Supplementary table 1.** Unadjusted and adjusted logistic regression models estimating the effect of the intervention on the proportion of residents with one or more regular PIMs. *CCI = Charlson Comorbidity Index, CI = Confidence Interval, PIMS = Potentially Inappropriate Medications.* *Adjusted model includes age, sex, presence/absence of a dementia diagnosis, CCI, number of regular medications, and presence/absence of concurrent NPS MedicineWise intervention*

| **Outcome: Resident’s ACB Scale** | **Unadjusted model Rate Ratio (95% CI)** | **p value** | **Adjusted model Rate Ratio (95% CI)** | **p value** |
| --- | --- | --- | --- | --- |
| **Control or Intervention arms** | | | | |
| Control | 1.00 | - | 1.00 | - |
| Intervention | 1.054 (0.857 - 1.298) | 0.617 | 1.017 (0.797 - 1.299) | 0.890 |
| **Timepoints** | | | | |
| Baseline | 1.00 | - | 1.00 | - |
| Endpoint | 0.942 (0.848 - 1.046) | 0.260 | 0.987 (0.888 - 1.098) | 0.809 |
| **Two-way interaction effects** | | | | |
| Intervention arm*Endpoint | **0.832 (0.705 - 0.981)** | **0.028** | **0.800 (0.678 - 0.944)** | **0.008** |
| **Age** | - | - | **0.982 (0.974 - 0.990)** | **<0.001** |
| **Sex** | | | | |
| Female | - | - | 1.00 | - |
| Male | - | - | 1.115 (0.975 - 1.274) | 0.112 |
| **Dementia diagnosis** | | | | |
| Residents without dementia diagnosis | - | - | 1.00 | - |
| Residents with dementia diagnosis | - | - | 1.012 (0.897 - 1.142) | 0.850 |
| **Total CCI** | - | - | **1.044 (1.010 - 1.079)** | **0.011** |
| **Number of regular medications** | - | - | **1.098 (1.085 - 1.112)** | **<0.001** |
| **NPS MedicineWise intervention** | | | | |
| No Intervention | - | - | 1.00 | - |
| Intervention | - | - | 1.122 (0.797 - 1.579) | 0.511 |

**Supplementary table 2**. Unadjusted and adjusted Poisson regression models estimating the effect of the intervention on residents’ ACB score. *CCI = Charlson Comorbidity Index, CI = Confidence Interval, PIMS = Potentially Inappropriate Medications. ACB = Anticholinergic Cognitive Burden. Adjusted model includes age, sex, presence/absence of a dementia diagnosis, CCI, number of regular medications, and presence/absence of concurrent NPS MedicineWise intervention.*

| **Outcome: Proportion of residents with at least one regular benzodiazepine or antipsychotic** | **Unadjusted model Odds Ratio (95% CI)** | **p value** | **Adjusted model Odds Ratio (95% CI)** | **p value** |
| --- | --- | --- | --- | --- |
| **Control or Intervention arms** |  |  |  |  |
| Control | 1.00 | - | 1.00 | - |
| Intervention | 0.999 (0.736 - 1.357) | 0.996 | 0.946 (0.628 - 1.424) | 0.790 |
| **Timepoints** |  |  |  |  |
| Baseline | 1.00 | - | 1.00 | - |
| Endpoint | 0.929 (0.711 - 1.213) | 0.587 | 1.022 (0.768 - 1.359) | 0.883 |
| **Two-way interaction effects time** |  |  |  |  |
| Intervention arm*Endpoint | 0.732 (0.487 - 1.101) | 0.134 | 0.676 (0.439 - 1.042) | 0.076 |
| **Age** | - | - | **0.933 (0.918 -0.948)** | **<0.001** |
| **Sex** |  |  |  |  |
| Female | - | - | 1.00 | - |
| Male | - | - | 0.938 (0.719 - 1.224) | 0.637 |
| **Dementia diagnosis** |  |  |  |  |
| Residents without dementia diagnosis | - | - | 1.00 | - |
| Residents with dementia diagnosis | - | - | **1.729 (1.339 - 2.233)** | **<0.001** |
| **Total CCI** | - | - | 0.941 (0.874 - 1.013) | 0.108 |
| **Number of regular medications** | - | - | **1.145 (1.115 - 1.177)** | **<0.001** |
| **NPS MedicineWise intervention** |  |  |  |  |
| No Intervention | - | - | 1.00 | - |
| Intervention | - | - | 1.243 (0.733 - 2.107) | 0.419 |

**Supplementary table 3.** Unadjusted and adjusted logistic regression models estimating the effect of the intervention on the proportion of residents with one or more regular benzodiazepine or antipsychotic. *CCI = Charlson Comorbidity Index, CI = Confidence Interval. Adjusted model includes age, sex, presence/absence of a dementia diagnosis, CCI, number of regular medications, and presence/absence of concurrent NPS MedicineWise intervention.*

| **Outcome: Chlorpromazine** **equivalent daily dose (mg) per resident** | **Unadjusted model coefficient (95% CI)** | **p value** | **Adjusted model coefficient (95% CI)** | **p value** |
| --- | --- | --- | --- | --- |
| **Control or Intervention arms** |  |  |  |  |
| Control | 1.00 | - | 1.00 | - |
| Intervention | -0.120 (-0.345 - 0.106) | 0.298 | 0.006 (-0.204 - 0.217) | 0.953 |
| **Timepoints** |  |  |  |  |
| Baseline | 1.00 | - | 1.00 | - |
| Endpoint | -0.015 (-0.141 - 0.111) | 0.817 | -0.002 (-0.129 - 0.125) | 0.971 |
| **Two-way interaction effects** |  |  |  |  |
| Intervention arm*Endpoint | -0.198 (-0.405 - 0.008) | 0.060 | **-0.250 (-0.456 - -0.043)** | **0.018** |
| **Age** | - | - | **-0.023 (-0.034 - -0.012)** | **<0.001** |
| **Sex** |  |  |  |  |
| Female | - | - | 1.00 | - |
| Male | - | - | -0.113 (-0.283 - 0.056) | 0.190 |
| **Dementia diagnosis** |  |  |  |  |
| Residents without dementia diagnosis | - | - | 1.00 | - |
| Residents with dementia diagnosis | - | - | **-0.192 (-0.357 - -0.027)** | **0.022** |
| **Total CCI** | - | - | 0.021 (-0.025 - 0.067) | 0.363 |
| **Number of regular medications** | - | - | 0.016 (<-0.001- 0.032) | 0.050 |
| **NPS MedicineWise intervention** |  |  |  |  |
| No Intervention | - | - | 1.00 | - |
| Intervention | - | - | -0.151 (-0.418 -0.116) | 0.267 |

**Supplementary table 4**. Unadjusted and adjusted Gamma regression models estimating the effect of the intervention on the chlorpromazine equivalent daily dose (mg) per resident. *CCI = Charlson Comorbidity Index, CI = Confidence Interval, mg= milligrams. Adjusted model includes age, sex, presence/absence of a dementia diagnosis, CCI, number of regular medications, and presence/absence of concurrent NPS MedicineWise intervention.*

| **Outcome: Diazepam equivalent daily dose (mg) per resident** | **Unadjusted model coefficient (95% CI)** | **P value** | **Adjusted model coefficient (95% CI)** | **p value** |
| --- | --- | --- | --- | --- |
| **Control or Intervention arms** |  |  |  |  |
| Control | 1.00 | - | 1.00 | - |
| Intervention | 0.046 (-0.195 - 0.288) | 0.707 | -0.111 (-0.308 - 0.085) | 0.265 |
| **Timepoints** |  |  |  |  |
| Baseline | 1.00 | - | 1.00 | - |
| Endpoint | -0.118 (-0.320 - 0.084) | 0.250 | 0.079 (-0.172 - 0.330) | 0.535 |
| **Two-way interaction effects** | | | | |
| Intervention arm*Endpoint | -0.093 (-0.400 - 0.214) | 0.551 | -0.129 (-0.428 - 0.170) | 0.397 |
| **Age** | - | - | -0.008 (-0.017 - 0.002) | 0.103 |
| **Sex** |  |  |  |  |
| Female | - | - | 1.00 | - |
| Male | - | - | -0.073 (-0.236 - 0.090) | 0.377 |
| **Dementia diagnosis** |  |  |  |  |
| Residents without dementia diagnosis | - | - | 1.00 | - |
| Residents with dementia diagnosis | - | - | -0.100 (-0.252 - 0.052) | 0.198 |
| **Total CCI** | - | - | -0.016 (-0.056 - 0.023) | 0.417 |
| **Number of regular medications** | - | - | 0.004 (-0.009 - 0.017) | 0.536 |
| **NPS MedicineWise intervention** | | | | |
| No Intervention | - | - | 1.00 | - |
| Intervention | - | - | -0.047 (-0.397 - 0.302) | 0.790 |

**Supplementary table 5**. Unadjusted and adjusted Gamma regression models estimating the effect of the intervention on the diazepam equivalent daily dose (mg) per resident. *CCI = Charlson Comorbidity Index, CI = Confidence Interval, mg= milligrams. Adjusted model includes age, sex, presence/absence of a dementia diagnosis, CCI, number of regular medications, and presence/absence of concurrent NPS MedicineWise intervention.*

| **Outcome: Proportion of residents with complete ADR documentation** | **Unadjusted model Odds Ratio (95% CI)** | **p value** | **Adjusted model Odds Ratio (95% CI)** | **p value** | |
| --- | --- | --- | --- | --- | --- |
| **Control or Intervention arms** |  |  |  |  | |
| Control | 1.00 | - | 1.00 | - | |
| Intervention | 0.766 (0.460 - 1.276) | 0.305 | 0.813 (0.473 - 1.398) | 0.453 | |
| **Timepoints** |  |  |  |  | |
| Baseline | 1.00 | - | 1.00 | - | |
| Endpoint | 1.316 (0.760 - 2.278) | 0.326 | 1.343 (0.776 - 2.327) | 0.292 | |
| **Two-way interaction effects** |  |  |  |  | |
| Intervention arm*Endpoint | 1.127 (0.518 - 2.450) | 0.763 | 1.109 (0.510 - 2.408) | 0.794 | |
| **Age** | - | - | 1.007 (0.982 - 1.032) | 0.608 | |
| **Sex** |  |  |  |  | |
| Female | - | - | 1.00 | - | |
| Male | - | - | 0.845 (0.562 - 1.271) | 0.420 | |
| **Dementia diagnosis** |  |  |  |  | |
| Residents without dementia diagnosis | - | - | 1.00 | - | |
| Residents with dementia diagnosis | - | - | 0.936 (0.628 - 1.395) | 0.744 | |
| **Total CCI** | - | - | 1.008 (0.895 - 1.135) | 0.897 | |
| **Number of regular medications** | - | - | 1.025 (0.981 - 1.072) | 0.271 |  |
| **NPS MedicineWise intervention** |  |  |  |  | |
| No Intervention | - | - | 1.00 | - | |
| Intervention | - | - | 0.757 (0.415 - 1.381) | 0.364 | |

**Supplementary table 6**. Unadjusted and adjusted logistic regression models estimating the effect of the intervention on the proportion of residents with complete ADR documentation. *ADR = Adverse Drug Reaction, CCI = Charlson Comorbidity Index, CI = Confidence Interval. Adjusted model includes age, sex, presence/absence of a dementia diagnosis, CCI, number of regular medications, and presence/absence of concurrent NPS MedicineWise intervention.*

| **Outcome: Number of regular medicines per resident** | **Unadjusted model Rate Ratio (95% CI)** | **p value** | **Adjusted model Rate Ratio (95% CI)** | **p value** |
| --- | --- | --- | --- | --- |
| **Control or Intervention arms** |  |  |  |  |
| Control | 1.00 | - | 1.00 | - |
| Intervention | 1.034 (0.927 - 1.154) | 0.547 | 1.029 (0.974 - 1.087) | 0.558 |
| **Timepoints** |  |  |  |  |
| Baseline | 1.00 | - | 1.00 | - |
| Endpoint | 0.941 (0.907 - 0.976) | **0.001** | **0.930 (0.896 - 0.965)** | **<0.001** |
| **Two-way interaction effects** | | | | |
| Intervention arm*Endpoint | 1.033 (0.978 - 1.092) | 0.243 | 1.029 (0.974 - 1.087) | 0.313 |
| **Age** | - | - | **0.997 (0.994 - 1.000)** | **0**.**043** |
| **Sex** |  |  |  |  |
| Female | - | - | 1.00 | - |
| Male | - | - | 0.968 (0.925 - 1.013) | 0.158 |
| **Dementia diagnosis** | | | | |
| Residents without dementia diagnosis | - | - | 1.00 | - |
| Residents with dementia diagnosis | - | - | **0.857 (0.823 - 0.892)** | **<0.001** |
| **Total CCI** | - | - | **1.040 (1.028 - 1.051)** | **<0.001** |
| **NPS MedicineWise intervention** | | | | |
| No Intervention | - | - | 1.00 | - |
| Intervention | - | - | 1.019 (0.875 - 1.187) | 0.805 |

**Supplementary table 7**. Unadjusted and adjusted Poisson regression models estimating the effect of intervention on the number of regular medicines per resident. *CCI = Charlson Comorbidity Index, CI = Confidence Interval. Adjusted model includes age, sex, presence/absence of a dementia diagnosis, CCI, and presence/absence of concurrent NPS MedicineWise intervention.*
